# A mutation in the brassinosteroid biosynthesis gene *CpDWF5* disrupts vegetative and reproductive development and the salt stress response in squash (*Cucurbita pepo*)

**DOI:** 10.1093/hr/uhae050

**Published:** 2024-02-23

**Authors:** Sonsoles Alonso, Gustavo Cebrián, Keshav Gautam, Jessica Iglesias-Moya, Cecilia Martínez, Manuel Jamilena

**Affiliations:** Department of Biology and Geology, Agrifood Campus of International Excellence (CeiA3), and Research Center CIAMBITAL, University of Almería, Ctra. Sacramento s/n, 04120 Almería, Spain; Department of Biology and Geology, Agrifood Campus of International Excellence (CeiA3), and Research Center CIAMBITAL, University of Almería, Ctra. Sacramento s/n, 04120 Almería, Spain; Department of Biology and Geology, Agrifood Campus of International Excellence (CeiA3), and Research Center CIAMBITAL, University of Almería, Ctra. Sacramento s/n, 04120 Almería, Spain; Department of Biology and Geology, Agrifood Campus of International Excellence (CeiA3), and Research Center CIAMBITAL, University of Almería, Ctra. Sacramento s/n, 04120 Almería, Spain; Department of Biology and Geology, Agrifood Campus of International Excellence (CeiA3), and Research Center CIAMBITAL, University of Almería, Ctra. Sacramento s/n, 04120 Almería, Spain; Department of Biology and Geology, Agrifood Campus of International Excellence (CeiA3), and Research Center CIAMBITAL, University of Almería, Ctra. Sacramento s/n, 04120 Almería, Spain

## Abstract

A *Cucurbita pepo* mutant with multiple defects in growth and development has been identified and characterized. The mutant *dwfcp* displayed a dwarf phenotype with dark green and shrinking leaves, shortened internodes and petioles, shorter but thicker roots and greater root biomass, and reduced fertility. The causal mutation of the phenotype was found to disrupt gene *Cp4.1LG17g04540*, the squash orthologue of the *Arabidopsis* brassinosteroid (BR) biosynthesis gene *DWF5*, encoding for 7-dehydrocholesterol reductase. A single nucleotide transition (G > A) causes a splicing defect in intron 6 that leads to a premature stop codon and a truncated CpDWF5 protein. The mutation co-segregated with the dwarf phenotype in a large BC_1_S_1_ segregating population. The reduced expression of *CpDWF5* and brassinolide (BL) content in most mutant organs, and partial rescue of the mutant phenotype by exogenous application of BL, showed that the primary cause of the dwarfism in *dwfcp* is a BR deficiency. The results showed that in *C. pepo*, *CpDWF5* is not only a positive growth regulator of different plant organs but also a negative regulator of salt tolerance. During germination and the early stages of seedling development, the dwarf mutant was less affected by salt stress than the wild type, concomitantly with a greater upregulation of genes associated with salt tolerance, including those involved in abscisic acid (ABA) biosynthesis, ABA and Ca^2+^ signaling, and those coding for cation exchangers and transporters.

## Introduction

Plant architecture is one of the most relevant agronomic traits that affect plant photosynthesis and nutrient distribution, which finally has a great impact on crop yield. The introduction of dwarfing genes into cereal crops was crucial to enhance lodging resistance and improve the harvest index in the so-called Green Revolution. Dwarf varieties, such as the Micro-Tom tomato, are also model cultivars for functional genomics research [1]. Therefore, understanding the genetic mechanisms of plant height is important for plant breeding.

Numerous dwarf mutants have been identified in many species. Most of the identified mutations affect the biosynthesis or signal transmission process of phytohormones, such as gibberellins (GAs), auxins [indole-3-acetic acid (IAA)], ethylene (ETH), strigolactone, and brassinosteroids (BRs). Among the dwarf mutants related to GAs, it is worth mentioning the mutants *anther ear1* (*an1*), *dwarf1* (*d1*), *d3*, and *d5* in maize [2–5]; *gid1* in rice [6]; *CsCLAVATA1* in cucumber [7]; or *rht1* in wheat [8]. Other dwarf mutants, including *VT2*, *Brachytic2*, and *ZmPIN1a* in maize [9–11] and mutants *yuc1D*, *pin*, and *pgp* in *Arabidopsis* [12–14], were reported to be defective in IAA biosynthesis or polar transport. The dwarf mutant *ZmACS7* from maize is defective in ACC synthase 7, a key enzyme in ETH biosynthesis [15]. Strigolactone-related dwarf mutants, such as *d27*, *d14*, *d53*, or *d27*, have also been identified in rice [16–18].

The role of BRs in plant growth and development is also essential. Currently, it is known that BRs have a key role not only in cell elongation but also in cell division, photomorphogenesis, xylem differentiation, plant reproduction, and responses to abiotic and biotic stress [19]. Numerous mutants related to BR biosynthesis or perception have been isolated in *Arabidopsis*, tomato, rice, cucumber, barley, and pea. The mutants *cpa*, *scp-1*, and *scp-2* in cucumber [20–22]; *dwf1*, *cpd/dwf3*, *dwf4*, *dwf5*, *det2/dwf6*, *stel/dwf7*, *dwf12*, and *bri1* in *Arabidopsis* [23–30]; *Ika* and *Ikb* in pea [31]; *d*, *d^X^*, *dpy*, *cu3*, and *abs* in tomato [32–34]; *na1*, *na2*, and *brd1* in maize [35–37]; or *dwarf2*, *dwarf11*, *d61*, and *brd1 in* rice [38–41] show a dwarf phenotype associated with a defect in BR biosynthesis or signaling pathways.

Mutants with lesions in intermediates or key enzymes involved in the biosynthesis of sterols, the precursors of BRs, also show a similar dwarf phenotype, including the *Arabidopsis* mutants *dwf1*, *dwf5*, and *dwf7* [28, 29, 42]. Mutation *dwf1* was identified as a defective flavin adenine dinucleotide (FAD)-dependent oxidoreductase that catalyzes the reduction of C-24 [42], while *Arabidopsis dwf7* and *dwf5* were reported to be deficient in the Δ7-sterol-C-5 desaturase [28] and sterol Δ7-reductase (S7R) [29], respectively. The phenotype of the *Arabidopsis* S7R-disrupted mutant *dwf5* includes abnormal growth and development with small and dark green leaves, short stems, pedicels, and petioles [29].

No genes related to the biosynthesis of sterol and BRs have been reported in *Cucurbita pepo* (*C. pepo)*. In this study, the identification and characterization of the squash mutant *dwfcp* are reported. The mutant displays a dwarf phenotype, affected in plant height, in the length of all vegetative and reproductive organs, and in fertility. However, the mutant shows thicker roots and higher root biomass, as well as enhanced tolerance to salt stress during earlier stages of plant development. The causal mutation of the phenotype was found to disrupt the gene *Cp4.1LG17g04540*, the squash orthologue of the *Arabidopsis* BR biosynthesis gene *DWF5*, encoding for 7-dehydrocholesterol reductase (S7R).

## Results

### Phenotypic characterization of the *dwfcp* mutant

A dwarf mutant was isolated from a *C. pepo* ethyl methanesulfonate (EMS) mutant collection. The so-called *dwfcp* mutant was backcrossed twice with MUCU16, the background genotype, and then selfed. The wild-type (WT) and mutant phenotypes were found in the resulting BC_1_S_1_ and BC_2_S_1_ segregating populations in a 3:1 ratio (Supplemental Table S1), indicating that the mutation is recessive. BC_2_S_1_ plants were used for phenotyping because the backcrosses are able to remove EMS mutations other than the selected *dwfcp*.

**Figure 1:**
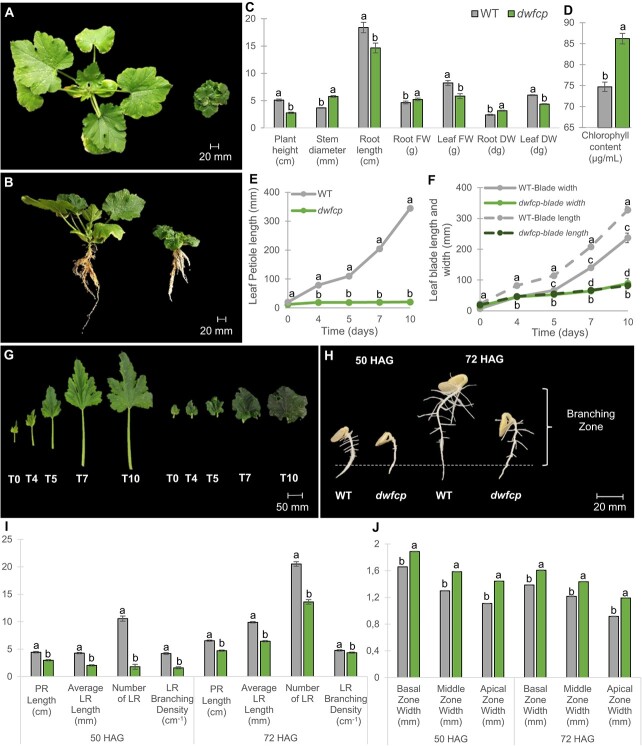
Phenotypic characterization of the *dwfcp* mutant. Phenotype comparison between WT (left) and *dwfcp* (right) seedlings at the stage of 7 true leavess. (A) Top view and (B) side view. (C) Comparison of plant height, stem diameter, root length, root biomass, and leaf biomass between WT and *dwfcp* at 7 true leaves seedling stage. (D) Chlorophyll content in WT and *dwfcp* leaves. (E) Growth rate of WT and *dwfcp* leaf petiole over time. (F) Growth rate of WT and *dwfcp* leaf blade over time. (G) Leaf development in WT (left) and mutant (right) plants. (H) Root growth and development during the establishment of WT and *dwfcp* seedlings (at 50 and 72 HAG). (I) Statistical comparison of parent root (PR) and LR length, number of LR, and LR branching density between WT and *dwfcp* at 50 and 72 HAG. (J) Width of the PR in three different zones (basal, middle, and apical) during the establishment of WT and mutant seedlings at 50 and 72 HAG. The LR branching density (cm^−1^) was calculated as the number of emerged LR per unit length of the root branching zone (in centimeters). The root branching zone includes the zone of the parent root that extends from the most rootward emerged LR to the shoot base. Error bars represent SE. Different letters indicate significant differences (*P* ≤ 0.05).

The mutant had a clear dwarf phenotype during vegetative development (Fig. 1A and B). The mutant plants showed shorter but wider stems, smaller, wrinkled, dark green leaves, and shorter petioles (Fig. 1A–F). The roots were also shorter but showed higher fresh weight (FW) and dry weight (DW) than those of the WT (Fig. 1C). However, the FW and DW of the aerial part of the plant were reduced in *dwfcp* compared to WT (Fig. 1C). The leaf chlorophyll content was significantly higher in the mutant than in the WT (Fig. 1D), which would explain the darker green color of the mutant plants.

The growth rate of the WT and *dwfcp* leaves is shown in Fig. 1E–G. The growth of the leaf petioles, but also the longitudinal and lateral growth of the leaf blade, was significantly lower in the mutant than in the WT. The petioles of the WT leaves grew an average of 35 cm in 10 days, while those of the *dwfcp* barely grew 1 cm during the same period of time (Fig. 1E). In the WT leaf blade, the longitudinal growth rate was greater than the transverse growth rate, resulting in leaves whose length was greater than their width. However, the longitudinal growth rate and transverse growth rate of the *dwfcp* leaf blade were similar, resulting in the development of round leaves (Fig. 1F and G). Scanning electron microscopy (SEM) analysis of WT and mutant stems indicated that cell size was significantly shorter but wider in *dwfcp* than in the WT (Supplemental Fig. S1), indicating that the dwarf phenotype is caused by a reduction in cell elongation.

The development of parental and lateral roots was compared in WT and *dwfcp* seedlings (Fig. 1H–J). Both the parent root and the lateral roots (LR) were shorter in *dwfcp* than in WT, and the branching density of LR was also lower in *dwfcp* seedlings at both 50 and 72 h after germination (Fig. 1H and I). The width of the basal, middle and apical zones of the parent root was found to be greater in the mutant than in the WT, which may explain the higher biomass of mutant roots (Fig. 1H–J). These results indicate that BRs play a positive role in the formation and development of the lateral root in *C. pepo*.

**Figure 2:**
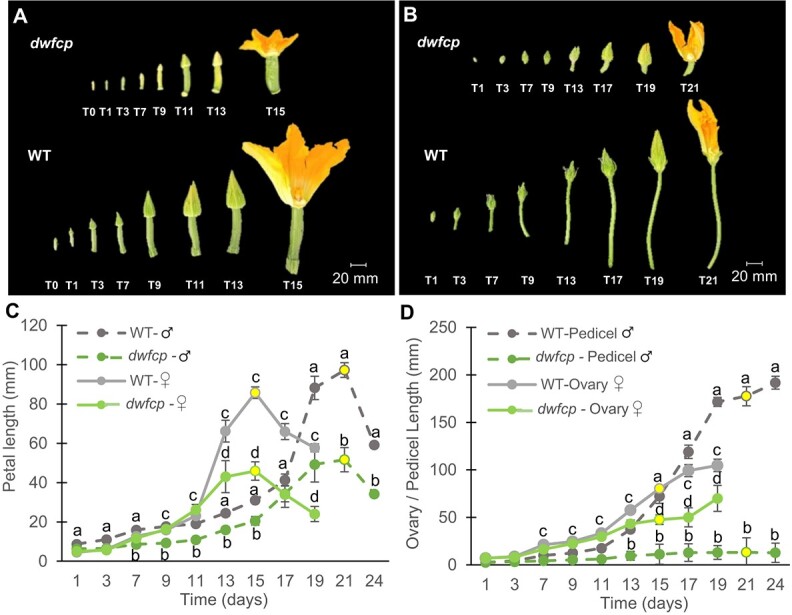
Effect of the *dwfcp* mutation on floral development. (A) Comparison of the growth of WT and *dwfcp* female flowers and (B) male flowers. (C) Growth rate of WT and *dwfcp* corolla over time. (D) Comparison between genotypes of the ovary and pedicel growth rate of female and male flowers, respectively, during a period of 24 days. The growth of flower ovaries was recorded every 2 days for 24 days starting when they were <1 cm length. The time-points at which >80% of the flowers reached anthesis are indicated in yellow. Error bars represent SE. Different letters indicate significant differences (*P* ≤ 0.05).

The mutation *dwfcp* affected floral development (Fig. 2). The growth rate of the *dwfcp* corolla was lower than that of WT (Fig. 2A–C). The anthesis time, the period of time taken for a <1 cm floral bud to develop and open, was earlier in female flowers (15 days) than in male flowers (21 days), both in the WT and *dwfcp* plants, and no significant differences were found between phenotypes (Fig. 2C and D). Significant differences were also detected in the growth rates of the WT and *dwfcp* ovaries and fruits of the female flowers, and in the pedicels of the male flowers (Fig. 2D). WT and *dwfcp* ovaries/fruits showed a similar growth rate during the first 11 days. After that time, the ovaries of the WT continued to grow, while mutant ovaries slowed or even stopped their growth. At anthesis, the ovary of the WT reached ~8 cm in length, while the *dwfcp* ovary reached ~5 cm in length.

The female flowers of *dwfcp* were pollinated with WT MUCU16 pollen, but most fruits aborted or were unable to set seeds, indicating a lack of female fertility in the mutant. However, male *dwfcp* flowers produced pollen and were able to plant fruit and seeds when used as pollinators of the background genotype MUCU16 and then selfed to obtain BC_1_S_1_ generations, although not all crosses performed were successful, also indicating a reduction in male fertility in the *dwfcp* male flowers compared to the WT. No differences were found in the size or weight of WT and *dwfcp* seeds from a BC_2_S_1_ segregating population (Supplemental Fig. S2).

### *dwfcp* is a mutation in the 3′ splicing site of *CpDWF5* intron 6

**Figure 3:**
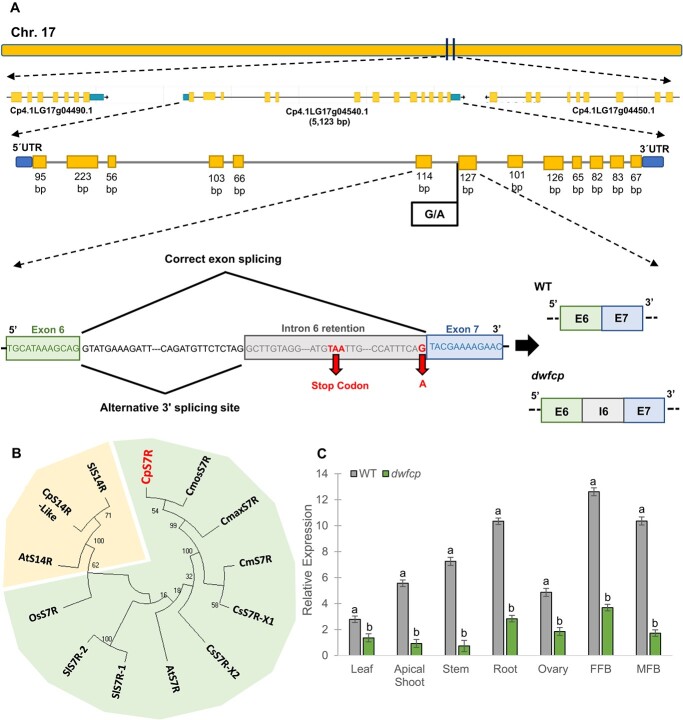
Identification and characterization of the *DWFCP* locus. (A) Identification of *dwfcp* causal mutation by BSA-sequencing. The *DWFCP* locus corresponds to gene *Cp4.1LG17g04540.1* (*CpDWF5*), located in the chromosome 17. The mutation produces a 3′-splice site mutation of intron 6, a premature stop codon and a truncated CpDWF5 protein. (B) Phylogenetic tree of sterol Δ^7^ reductases (S7R) and sterol Δ^14^ reductases (S14R) from different cucurbit species, *C. maxima*, *C. melo, Cucumis sativus*, *C. moschata*, and *C. pepo*, together with those of the most studied model species, *A. thaliana*, *O. sativa*, and *S. lycopersicum*. Bootstrap values for the main branches are depicted on the tree. (C) Relative gene expression of *CpDWF5* in different tissues of WT and *dwfcp* plants measured by qRT-PCR. FFB, female floral bud excluding the ovary; MFB, male floral bud. Error bars represent SE. Different letters indicate significant differences (*P* ≤ 0.05).

A bulked segregant analysis sequencing (BSA-seq) approach was used to find the causal mutation of *dwfcp*. Thirty WT plants and thirty dwarf plants from a BC_1_S_1_ segregating population were used to construct two DNA bulks. Around 80 million reads in each bulk (>98% of the sequencing reads, representing an average depth of 44.49) were mapped against the *C. pepo* reference genome (version 4.1), which allowed the identification of more than 400 000 single nucleotide polymorphisms (SNPs) in each bulk (Table 1). The following parameters were established for SNP filtering: (i) transitions G > A and C > T, (ii) alternative allele frequency (AF) = 1 in mutant bulk, and (iii) SNPs with non-synonymous mutations. As the phenotype of WT and heterozygous plants is indistinguishable, we were more flexible with the filter on the AF of the WT bulk. Thus, after filtering for AF ≤ 0.6 in the WT bulk and AF = 1 in the mutant bulk, 1126 SNPs were identified, of which 317 were canonical EMS mutations (C > T and G > A). An SNP found on chromosome 17 was the only one with a significant impact on the protein (Table 1 and Fig. 3). SNP alleles were genotyped in 300 plants from a BC_1_S_1_ population for the putative mutation to prove that it was responsible for the *dwfcp* phenotype, and the results demonstrated 100% co-segregation between the mutant phenotype and the G > A mutation located on chromosome 17.

**TABLE 1:** Summary sequencing data for WT and *dwfcp*

Sequencing results			WT	*dwfcp*		
No. of reads			95 993 772	84 920 814		
Mapped reads (%)			98.09	98.28		
Average depth			44.49	39.45		
Coverage at least 4× (%)			95.37	94.86		
SNP filtering						
Total no. of SNPs			425 994	417 882		
AF (WT) ≤ 0.6; AF (*dwfcp*) = 1			1126	1126		
EMS SNPs G > A or C > T			317	317		
EMS SNPs (GQ > 75; DP > 20)			2	2		
High-impact SNPs			0	1		
Candidate SNPs						
Chr	Position	Ref.	Alt	Gene ID	Effect	Functional annotation
17	3.048.864	G	A	Cp4.1LG17g04540	Splice site mutation	7-Dehydrocholesterol reductase

DP, read depth; GQ, genotype quality.

The identified mutation affected a single nucleotide at the 3′ splicing site of the sixth intron of the gene *Cp4.1LG17g04540,* encoding for a 7-dehydrocholesterol reductase, an enzyme in the cholesterol and BR biosynthesis pathway. This gene was homologous to the *Arabidopsis DWF5* gene, so it was named *CpDWF5*. To see whether the EMS mutation disrupts intron 6 splicing, the WT and mutant gene transcripts were amplified by reverse transcription polymerase chain reaction (RT-PCR) and then sequenced. The mutant transcript had an additional fragment of 76 nucleotides that corresponded to part of the sequence of the sixth intron of the gene, which generates a premature stop codon and a truncated protein (Fig. 3A). Although the genome of *C. pepo* is duplicated [43], the gene *CpDWF5* on chromosome 17 does not have any other paralog in the genome. The gene encoded a putative protein of 435 amino acids. The protein sequence was used in BLAST searches against the NCBI protein database, which demonstrated a high identity with homologous proteins in cucurbits, including *Cucurbita moschata* (99.08%), *Cucurbita argyrosperma* (98.62%), *Cucurbita maxima* (98.39%), *Momordica charantia* (91.71%), or *Cucumis melo* (91.24%), but also with other crops, including legumes (85.51% homology with *Glycine max*), Solanaceae (82.56% homology with *Solanum lycopersicum*), or Cruciferae (85.28% homology with *Brassica oleracea*). By using the 7-dehydrocholesterol reductase (S7R) protein sequences from different cucurbit species, together with *Arabidopsis thaliana*, *Oryza sativa*, and *S. lycopersicum*, a multiple sequence alignment was conducted using the software Omega (Supplemental Fig. S3). A phylogenetic tree was then inferred using not only these S7R protein sequences but also some S14R (Fig. 3B). As expected, squash CpDWF5 clustered together with other S7R enzymes in other plants but separated from S14R.

The expression of *CpDWF5* in different WT and *dwfcp* tissues is shown in Fig. 3C. *CpDWF5* was found to be expressed in all tissues, although the transcript accumulated the most in the roots and flower buds. These results suggest that *CpDWF5* plays a crucial role in the development and growth of most tissues in zucchini squash. The transcription level of *CpDWF5* was significantly lower in the mutant than in the WT, suggesting that the mutation was able to downregulate the accumulation of the transcript in the different organs analyzed.

### Exogenous BR treatment can partially rescue the *dwfcp* phenotype

**Figure 4:**
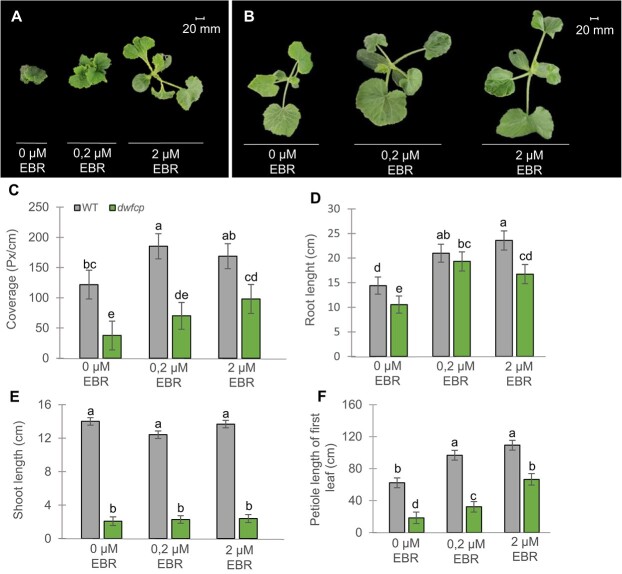
Partial rescue of *dwfcp* phenotype by exogenous application of EBR. (A–B) Top view of *dwfcp* (A) and WT (B) seedlings after 1 week of treatment with 0, 0.2, and 2 μM of EBR. (C–F) Effect of exogenous treatment with EBR on leaf coverage, and the length of shoots, roots, and leaf petioles of WT and *dwfcp* plants. Error bars represent SE from the treatments of nine plants per genotype and treatment. Different letters indicate significant differences between samples from different treatments and genotypes (*P* ≤ 0.05).

Since the *dwfcp* mutation affects the BR biosynthesis pathway, we wondered how an exogenous treatment with BRs would affect the mutant phenotype. WT and mutant seedlings were sprayed with two concentrations of epibrassinolide (EBR). Both WT and mutant seedlings responded to treatment, increasing leaf petiole, leaf blade growth (represented by leaf coverage parameter), and root length (Fig. 4C, D, and F). However, the length of shoots of WT or mutant plants was not affected by the treatments (Fig. 4E). The dosage effect on leaf growth was observed in the mutant but not in the WT plants (Fig. 4C), suggesting a deficiency of BRs in the mutant and hormone saturation in the WT under the most concentrated EBR treatment. Taken together, these results indicated that BRs were able to partially rescue the *dwfcp* phenotype, increasing the size of the leaf petiole and the leaf blade and the length of the root.

BR deficiency in *dwfcp* was also confirmed by measuring the content of brassinolide (BL) in different organs of WT and *dwfcp* plants. As expected, most of the organs analyzed, including the apical shoot, the leaf, and reproductive organs such as female and male flowers and the ovary, showed lower BL content in the mutant than in WT. However, the stems and roots of the WT and *dwfcp* plants did not show significant differences in BL content (Supplemental Fig. S4).

### Response of *dwfcp* to salt stress during germination and radicle growth

**Figure 5:**
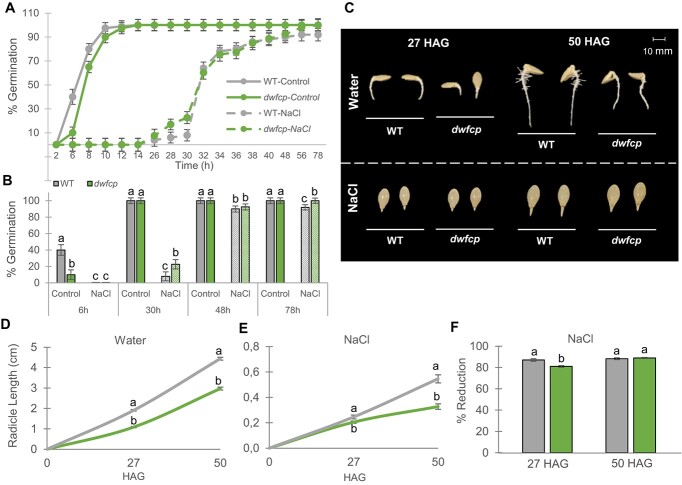
Effect of NaCl on the germination and radicle growth of WT and *dwfcp* seeds. (A) Germination rate of WT and *dwfcp* mutant seeds under control and saline conditions. (B) Effect of 200 mM NaCl on the germination percentage at 6, 30, 48, and 78 h. (C–E) Radicle length of WT and *dwfcp* seeds at different HAG under control and saline conditions. (F) Effect of salt on the percentage of reduction of radicle length. Error bars represent SE. Different letters indicate significant differences between samples (*P* ≤ 0.05).

To study the role of BRs in the germination of *C. pepo*, WT and *dwfcp* seeds were germinated under control (water) and salt conditions for 78 h, recording the germination as the time of radicle protrusion every 2 h (Fig. 5). Based on a dose–response study between NaCl concentration and germination stress on the seed germination of the genetic background of the mutant collection MUCU16 (Supplemental Fig. S5), a final concentration of 200 mM NaCl was selected for this assay since it led to a delay in germination of 1 day difference compared to control conditions, which would allow a clear distinction between sensitive and tolerant to salt stress phenotypes during seed germination.

In water, *dwfcp* germination was slightly delayed with respect to WT (Fig. 5A and B), indicating a positive role for BRs in the germination of *C. pepo.* However, the final percentage of germination was 100% for both genotypes. NaCl treatment delayed the initiation of germination in both WT and *dwfcp* compared to control conditions, although the mutant started to germinate earlier than WT and reached a higher percentage of germination under salinity conditions (Fig. 5A and B).

The effect of NaCl on the growth rate of WT and *dwfcp* radicle length was evaluated 27 and 50 h after germination (HAG) (Fig. 5C). The radicle length in water was always larger in the WT than in the mutant, both at 27 and 50 HAG (Fig. 5D), and the same was true under NaCl treatment (Fig. 5E), which was expected given the dwarf phenotype of the mutant. However, the percentage of reduction of the radicle length was significantly lower in *dwfcp* than in the WT at 27 HAG under saline conditions, which means that the *dwfcp* tolerated salinity better than the WT during this first stage of radicle development. At a later stage, 50 HAG, no significant differences were observed in the percentage of reduction of radicle length between WT and mutant (Fig. 5F).

### Response of *dwfcp* to salt stress during etiolation

**Figure 6:**
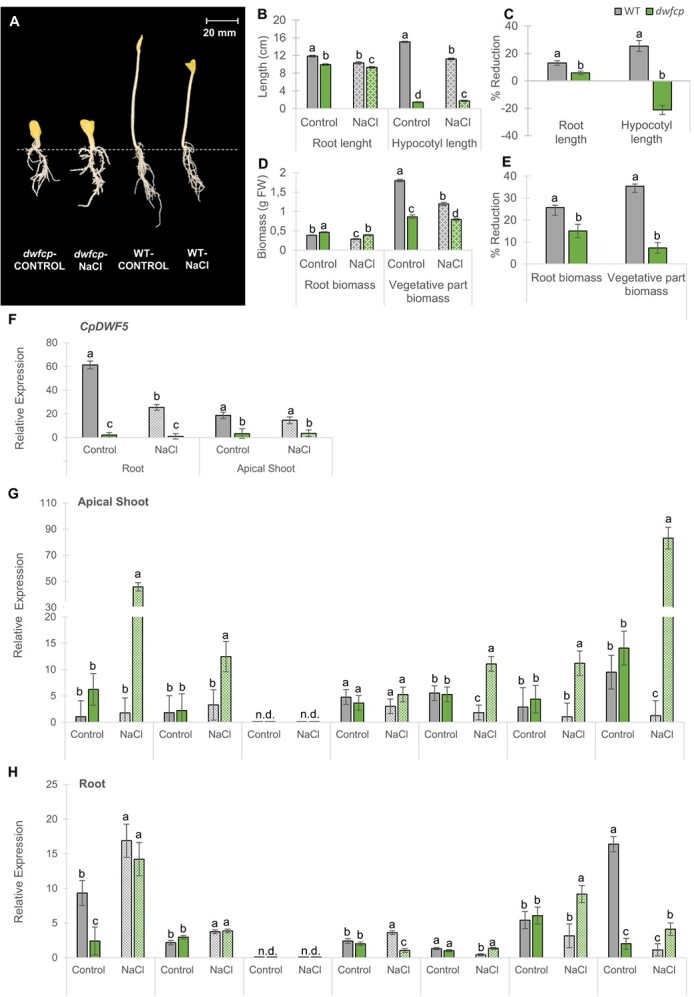
Effect of NaCl on growth parameters and gene expression of WT and *dwfcp* seedlings grown in darkness for 72 h. (A) WT and *dwfcp* seedlings under normal and saline conditions. (B) Effect of NaCl on root and hypocotyl length. (D) Effect of NaCl on root and vegetative part biomass. (C, E) Percentages of reduction of each parameter in response to salt. (F) Relative expression of *CpDWF5.* (G, H) Relative expression of genes involved in salt stress tolerance. N.d., not detected. Error bars represent SE. Different letters indicate statistically significant differences (*P* < 0.05) of the same gene expression between samples.

To study the effect of the BR-deficient mutation *dwfcp* of cell elongation, we measured seedling growth in darkness for 72 h under control and salinity conditions (Fig. 6). Similar to seed germination, a study of dose–response relationship between NaCl stress and seedling growth in line MUCU16 was carried out to select the optimal concentration for this assay (Supplemental Fig. S5). All saline concentrations (30, 60, 100, and 150 mM) reduced the analyzed parameters, except 60 mM NaCl, which did not reduce the hypocotyl length. This is why a concentration of 100 mM NaCl was finally selected, since it would allow a clear distinction between sensitive and tolerant-to-salinity phenotypes during seedling establishment.

The *dwfcp* mutant showed a de-etiolation phenotype, lacking the hypocotyl elongation observed in the WT-etiolated seedlings (Fig. 6A). NaCl treatment affected the length of the roots, as well as the biomass of the roots and aerial parts both in the WT and *dwfcp*, but the sensitivity of mutant seedlings to salt was lower than in the WT (Fig. 6B–D). Furthermore, though saline treatment reduced the length of hypocotyl in the WT, in the mutant it was greater than in control plants (Fig. 6B and C), meaning that the salt even stimulated the growth of mutant hypocotyl. In fact, under salt stress, the root length of the WT seedlings was reduced by ~13%, while the *dwfcp* mutant exhibited a reduction of only 5.8%. Hypocotyl length was reduced 25% in WT, while that of the mutant increased by 21% (Fig. 6C). In the same way, the biomass of the aerial part was reduced by 35% in WT, but only by 7% in the mutant (Fig. 6E). Lastly, the root biomass was higher in *dwfcp* than in the WT under control conditions (Fig. 6D), unlike the rest of the parameters, which is probably a consequence of the increased thickness of the mutant roots. As in the rest of the parameters analyzed, the reduction in root biomass was more pronounced in the WT (26%) than in the *dwfcp* (15%) (Fig. 6E). These data indicate that *dwfcp* was more tolerant to salinity than WT, not only during germination and radicle growth, but also during etiolation at seedling stage.

### Comparison of gene expression in WT and *dwfcp* in response to salt stress

To better understand the mechanisms that coordinate the enhanced salt tolerance of *dwfcp*, the expression of *CpDWF5*, but also genes related to salt tolerance in other species were compared in the apical shoots and roots of WT and *dwfcp* seedlings grown for 72 h in darkness under control and salinity conditions (Fig. 6F–H). The following genes were selected because they had already been reported to have a role in salinity tolerance in different species, including *C. pepo* [44]. *CpNCED2/5A* and *CpPP2C-A* are ABA biosynthesis and signaling genes, respectively, while *CpCYP707A2* is involved in ABA catabolism. *CpKEA4-2A* encodes for a K^+^/H^+^ efflux antiporter, *CpKUP6-1B* for a potassium transporter, and *CpNHK1-3B for a* Na^+^/H^+^ exchanger. The calcium signaling pathway is represented by the calmodulin-binding receptor-like cytoplasmic kinase gene *CpCRCK2B.*

In WT plants, the *CpDWF5* gene was expressed higher in the root than in the apical shoot, and it was significantly downregulated in response to salt treatment in the roots but not in the apical shoots (Fig. 6F). These data suggest that the response of *C. pepo* to salinity is associated with a reduction in the biosynthesis of BRs. As expected, the *dwfcp* mutant showed a very reduced expression of *CpDWF5* under both control and salinity stress (Fig. 6F).

Under control conditions, genes that in other species mediate the salt tolerance response, including genes that encode ion transporters, ABA biosynthesis, and ABA and Ca^2+^ signaling, showed a similar expression level in the apical shoots and roots of WT and *dwfcp* (Fig. 6G and H). However, under salt stress, some of these genes were expressed much higher in the mutant than in WT seedlings, especially in the apical shoot (Fig. 6G and H). This induction may explain the enhanced response of *dwfcp* to salt stress. In the apical shoot, K^+^ uptake permease *CpKUP6-1B gene*, Na^+^/H^+^ exchanger *CpNHK1-3B*, and the Ca^2+^ signaling gene for calmodulin-binding receptor-like cytoplasmic kinase *CpCRCK2B* were only upregulated by salt stress in *dwfcp* (Fig. 6G). The same was true for roots (Fig. 6H). Similarly, genes involved in the ABA biosynthesis and signaling pathway, including *CpNCED2/5A* and *CpPP2C-A*, were not regulated under salt stress in the WT apical shoot, while they were upregulated in the mutant (Fig. 6G). In roots, ABA genes were induced in both WT and *dwfcp* in response to salt stress, but no significant differences were found between genotypes (Fig. 6H). Of particular interest were the *CpNCED2/5A* and *CpCRCK2B* genes, whose expression was increased >45 and 80 times, respectively, in response to salinity in the apical shoot of *dwfcp* (Fig. 6G).

## Discussion

### BRs control vegetative and reproductive development in *C. pepo*

We have shown that the locus *DWFCP* corresponds to the gene *CpDWF5*, which encodes 7-dehydrocholesterol reductase (S7R), a key enzyme in the biosynthesis of sterols, the precursors of BRs [45]. Given the reduced level of BL (the most bioactive BR) in most of the *dwfcp* organs and the partial rescue of the mutant phenotype by exogenous application of BL, we concluded that the primary cause of the dwarfism in *dwfcp* is a deficiency in BRs. Furthermore, plant sterols are important structural components of cellular membranes and regulate membrane fluidity [46], and they also participate in cellulose biosynthesis [47]. This BR-independent role of sterols may also explain some of the phenotypic effects of *dwfcp* and why the *dwfcp* phenotype was only partially rescued by the application of BRs. Therefore, this mutant offers a novel genetic tool to study the effect of sterols and BRs in squash, a horticultural species of great importance.

The phenotypic effect of *dwfcp* on vegetative development, including shorter leaves, stems, pedicels, petioles, roots, and fruits, coincides with the defects reported in other BR-deficient mutants [27–29]. An increase in chlorophyll content and a darker green color was also found in *cpa*, a cucumber mutant similarly altered in an *S7R* gene [22]. BRs have been reported to maintain the stability of the thylakoid membrane and regulate chlorophyll molecules by controlling the activity of the chlorophyllase enzyme [48]. As in other BR mutants [49, 50], the shorter and wider roots of *dwfcp* indicate that BRs have an opposite effect on longitudinal and radial growth, and that the increased fresh and dry biomass of *dwfcp* roots is likely due to the increased thickness of the parental and lateral roots. The reduced branching of the mutant roots also suggests that BRs play a major role in lateral root initiation and development, possibly via its interaction with the auxin gradient in the root [51]. The BR-insensitive mutant *bri1* also displays a decreased number of lateral roots [52]. Understanding how BRs interact with other signals to regulate root growth and development in *C. pepo* represents a logical follow-up research target.

The reduced length of the *dwfcp* organs is due to a failure in the elongation of the cells. In fact, the number of cells in the mutant stem is greater than that in the WT, and the cells are shorter but wider than those of the WT, indicating that the *dwfcp* phenotype is caused by an alteration in cell size and shape. As in other BR mutants [22, 39, 53], this cell shape makes the width of leaf blades and roots, for example, not reduced nearly as much as their length, giving rise to rounder leaves and wider roots.

BRs also have a role in plant reproductive development [54]. *Arabidopsis* BR mutants *cpd*, *bin2*, and *bri1-201* have little or no male fertility due to reduced filament elongation or a failure in pollen tube elongation [24, 55, 56]. The male flowers of the *dwfcp* plants were less fertile than those of the WT, but not completely sterile. As in other BR mutants [57], we found that the seeds of *dwfcp* developed normally and germinated well. However, the slight delay in *dwfcp* germination compared to WT suggests that BRs may have a minor stimulating effect on the germination of *C. pepo.* Steber and McCourt [58] reported that *Arabidopsis* BR mutants *det2-1* and *bri1-1* germinate normally, but show increased sensitivity to ABA during germination and that BRs partially rescue the germination of GA-deficient and GA-insensitive mutants.

### *dwfcp* enhances tolerance to salt stress during earlier stages of plant development

Our physiological and molecular data have shown that sterols and BRs are involved in the squash salt stress response. The reduction in the germination rate, radicle growth, and hypocotyl and root elongation of seedlings in response to salt stress was found to be lower in *dwfcp* than in WT, suggesting that the mutant exhibits a higher tolerance to salinity than the WT, especially at early stages of seedling development growing in darkness. These are novel results indicating that sterols and BRs would play a negative role in *C. pepo* salt tolerance, at least under the tested conditions. Furthermore, ABA and Ca^2+^ biosynthesis and signaling pathways can mediate the tolerance response of *dwfcp* to salt, since the *dwfcp* mutation upregulated some of the genes involved in these two pathways, which are already known to control salt tolerance in *C. pepo* and other species [44]. The expression of ion transport genes, as well as the influx of Ca^2+^ in guard cells that leads to closure of the stomata, is known to be controlled by ABA [59, 60], but also ion homoeostasis and osmotic adjustment under salt stress [61]. We found that the *KUP6-1B* gene, which has a high affinity with the *Arabidopsis KUP6* gene, an ABA-responsive K^+^ transporter [60], was upregulated in the apical shoot and root of *dwfcp* exposed to salt. This large KUP/HAK/KT family of high-affinity K^+^ transporters was reported to function in the acquisition and translocation of K^+^ from roots to shoots and facilitates K^+^ efflux from the vacuole to regulate osmotic adjustment [60, 62].

The negative role of *CpDWF5* and BRs in salt tolerance was not previously reported. Exogenous BRs have been shown to mitigate the negative effects of salinity in many plants [63–65], and the *Arabidopsis* BR mutants *det2–1* and *bin2–1* were more sensitive to salt stress than the WT at germination and early seedling growth, suggesting that endogenous BRs play a positive role in salt tolerance [66]. However, recent studies have revealed a hormonal stress level-dependent biphasic effect (SLDB), whereby both the level of BRs and the level of stress can have an adverse effect on the tolerance of the plant to salt [67]. Furthermore, environmental factors such as temperature and light can also affect hormone signaling and transcriptional responses [68]. Given that our germination and seedling etiolation assays were performed under dark conditions and at a single NaCl concentration, we cannot exclude the possibility that the *dwfcp* mutant may respond differentially to other environmental and NaCl conditions. Alternatively, it is also possible that the higher tolerance of *dwfcp* to NaCl is not dependent on BRs, but on changes in the sterol profile, which is known to also regulate the plant response to abiotic stresses [69, 70]. Future studies determining the response of *dwfcp* to different doses of stress and environmental conditions will help to better understand the role of *CpDWF5* gene and BRs in *C. pepo* salt tolerance.

## Materials and methods

### Plant materials

In this study, we present the identification and characterization of a novel dwarf mutant line called *dwfcp*. It was identified from a direct phenotypic screening of a *C. pepo* EMS collection [71]. The M_2_ plants of the collection lines were planted and evaluated under standard greenhouse conditions. BC_1_S_1_ and BC_2_S_1_ segregating populations from the crosses of *dwfcp* (male) with the background genotype MUCU16 (female) and their subsequent self-pollination were developed for phenotyping and gene mapping.

### Phenotypic characterization of *dwfcp*

For the characterization and morphological identification of the *dwfcp* line, the weight and height of the plant, the diameter of the stem, as well as the growth of the vegetative and reproductive organs, the color of the leaf, and the cell size were evaluated in seedlings at the 7- leaf stage. The measurement of chlorophyll content was carried out on second true leaf samples according to the Warren procedure [72], and the calculation formula was based on the method described by Ritchie [73]. The analysis was conducted in three biological replicates per genotype and three plants per replicate. In addition, three technical replicates of each biological replicate were realized. Samples (5-mm sections from leaves of 7- leaf stage seedlings) for SEM were fixed in FAE (formaldehyde 3.7%: acetic acid 5%: absolute ethanol 50%), dehydrated through a graded ethanol series, critical point dried with liquid carbon dioxide in a pressure chamber BAL-TEC CPD 030 (Central Research Services, UAL, Spain), sputter coated with gold in a LEICA EM ACE 200 (Central Research Services, UAL, Spain), and photographed with a Zeiss Sigma 300 VP High-Resolution Field Emission Scanning Electron Microscope (Central Research Services, UAL, Spain).

The growth rates of vegetative and reproductive organs of WT and *dwfcp* plants were assessed by measuring the length of leaves, ovaries, and petals in both male and female flowers every 2 days for a total of 10 days in leaves or 24 days in flowers. A total of 10 leaves—15 male and 15 female flowers of each genotype—were evaluated, starting with leaves <3 cm and flower buds <1 cm in length. The anthesis time was estimated as the number of days taken for a <1-cm female or male floral bud to reach anthesis. The size and weight of the WT and *dwfcp* seeds from a segregating BC_2_S_1_ population were also evaluated. After the evaluation of the parameters, seeds were germinated under standard conditions, and the identification of WT and mutant seeds was carried out after genotyping for the *dwfcp* mutation using individual DNA from radicles.

Finally, the growth and development of the roots during the establishment of the WT and *dwfcp* seedlings were evaluated, including the width of the parent root in three different zones (basal, middle, and apical) and the formation and growth of the LR. ImageJ® was used to measure the elongation of both the parent and the LR of WT and *dwfcp* from digital images taken 50 and 72 h after the protrusion of the radicle (radicle length of ~1 mm). The LR branching density (cm^−1^) was calculated as the number of emerged LR per unit length in the root branching zone (in centimeters). The root branching zone includes the zone of the parent root that extends from the most rootward emerged LR to the shoot base. Since the seeds were selected from a BC_2_S_1_ population, the identification of WT and mutant seedlings was performed after genotyping for the *dwfcp* mutation using individual DNA from radicles.

### Identification of the causal mutation of *dwfcp* phenotype by whole genome resequencing

To identify the causal mutation of the *dwfcp* phenotype, 120 BC_1_S_1_ seeds were grown in trays until the seedling stage, where the phenotype was verified. Young leaves of 30 WT and 30 *dwfcp* plants were collected and pooled in two separate bulks. Genomic DNA was isolated using the CTAB method described previously [74] and then subjected to whole genome resequencing (WGS). The NEBNext® DNA Library Prep Kit (https://international.neb.com) was used to create a library of short fragments of ~350 bp randomly sheared from WT and mutant DNA bulks. Fragments were then briefly PCR-enriched with indexed oligos, and pair-end sequencing, with a read length of PE150 bp at each end, was performed into the Illumina® sequencing platform. The BWA software was used to align effective sequencing data with the reference genome of *C. pepo* v.4.1, and GATK HAPLOTYPECALLER and ANNOVAR were used to detect and annotate SNPs. Sequencing data from other lines of the EMS mutant squash collection were used to discard common variants between lines, as they represent variants already present in the background genotype MUCU16 before sequencing of the reference genome. The following parameters were taken into account to filter the sequencing data: [1] selection of EMS-induced canonical transitions C > T and G > A; [2] selection of variants with an alternative AF = 1 in the mutant bulk (*dwfcp/dwfcp*); [3] selection of SNPs with nonsynonymous mutations. Finally, Integrative Genomics Viewer (IGV) software and the Cucurbit Genomics Database (CuGenDB) were used to determine the impact of the final set of EMS SNPs on gene function (http://cucurbitgenomics.org/).

### Validation of the identified mutations by high-throughput genotyping of individual segregating plants

Kompetitive allele-specific PCR (KASP) technology was used to genotype 322 plants from a BC_1_S_1_ segregating population and to validate the causal mutation of the *dwfcp* phenotype. The LGC protocol and the FX96 Touch real-time PCR detection system (Bio-Rad®) were used to perform the KASP assay. LGC Genomics® synthesized the primers. A final reaction volume of 10 μl was used for multiplex PCRs, containing 5 μl KASP V4.0 2x Master Mix Standard ROX (LCG Genomics®), 0.138 μl KASP-by-Design Primer Mix (LCG Genomics®), and 4.862 μl of 20–50 ng/μl genomic DNA. SNP genotypes were finally identified using CFX Maestro™ software (Bio-Rad®).

### Sanger sequencing of *CpDWF5* cDNA in WT and *dwfcp*

Given that the *dwfcp* mutation was identified at the 3′ splicing site of the gene *CpDWF5*, the cDNA sequence of the gene in WT and *dwfcp* plants was compared. Total RNA from WT and *dwfcp* leaves was isolated according to the CTAB protocol. The cDNA RevertAid™ Kit (Thermo Fisher®) was used to convert the total RNA to cDNA. Templates for sequencing were amplified by PCR from cDNA (sequences of the forward and reverse primers in Supplemental Table S2) and NZYTaq II DNA polymerase (Nzytech®), according to the manufacturer’s protocol. A GeneJET PCR Purification Kit (Thermo Fisher®) was used to purify the PCR products and visualized on a gel. BigDye XTerminator™ Purification Kit (Thermo Fisher®) was used to purify the products of the sequencing reactions and the ABI 3500 Genetic Analyzer (Applied Biosystems®) to perform sequencing. Chromas software was used to analyze the sequencing results.

### Assessment of relative gene expression by quantitative RT-PCR

Transcript levels of the candidate gene *CpDWF5* were analyzed in different WT and *dwfcp* tissues, including male and female floral buds, ovaries, leaves, stems, apical shoots, and roots. Quantitative reverse transcription (qRT)-PCR assays were also performed to assess the relative expression of different genes involved in salt stress tolerance, both in the apical shoot and roots of WT and *dwfcp* seedlings grown under control and saline conditions for 72 h in darkness. The analyses were conducted in three biological replicates per genotype and growing condition, each derived from a pool of samples from five to six plants. Plant samples were collected and pulverized in liquid nitrogen, and total RNA was isolated according to the GeneJET Plant RNA Purification Kit (Thermo Fisher®) and converted into cDNA with the cDNA RevertAid™ Kit (Thermo Fisher Scientific®). qRT-PCR was performed in a 96-well plate using the CFX96 Touch Real-Time PCR Detection System Thermocycler (Bio-Rad®) in a 10 μl total volume with SYBR Green PCR Master Mix (BioRad®). The 2^-ΔΔCT^ method [75] was used to calculate gene expression values. Constitutive *CpEF1α* and *Cp18S* genes were used as reference genes to normalize the results of gene expression. For expression assessment in genes related to salt tolerance, the calibrator samples were those coming from plants under control conditions. For *CpDWF5* gene expression assessment, the calibrator sample was the one with the lowest transcription level. Supplemental Table S2 shows the sequences of the primers used for each qRT-PCR reaction.

### BR treatments

Given that the *dwfcp* mutation is in an enzyme of the BR biosynthesis pathway, the mutant phenotype was tried to be rescued by external BR application. Seeds were germinated in 96-well trays, and seedlings were transferred to pots and grown in a phytotron with 60% relative humidity (RH), 24°C, and a photoperiod of 16 h day/8 h night. The apical shoots of both WT and mutant plants were sprayed with 0, 0.2, and 2 μM EBR once a day for a week, since the plants had four true leaves. For hormone treatment, a stock solution of EBR (Sigma-Aldrich, CAS number: 78821-43-9) was prepared by dissolving 10 mg of EBR in *N*,*N*-dimethylformamide. Dilutions of the desired concentrations were prepared from the stock solution with distilled water. For control conditions, distilled water was used with an equivalent concentration of *N*,*N*-dimethylformamide. All solutions were supplemented with 0.1% Tween-20.

### Protein sequence alignment and phylogenetic analysis

The BLAST alignment tools at NCBI (http://www.blast.ncbi.nlm.nih.gov/) and Clustal Omega at EMBL-EBI (https://www.ebi.ac.uk/Tools/msa/clustalo/) were used to perform the alignments. The phylogenetic relations between different cucurbit species*, A. thaliana, O. sativa*, and *S. lycopersicum* S7R and sterol Δ^14^ reductase (S14R) proteins were assessed. Protein sequences were aligned using MEGA X software. The construction of phylogenetic trees was performed using MUSCLE and the maximum likelihood method based on the Poisson correction model, with 2000 bootstrap replicates. Protein sequences (Supplemental Table S3) used for multiplex alignment and phylogenetic study were obtained from the Arabidopsis Information Resource (https://www.arabidopsis.org/) and the Cucurbit Genomics Database (CuGenDB, http://cucurbitgenomics.org/). Gene Structure Display Server (GSDS) was used to visualize the *CpDWF5* gene structure, including the number and position of introns and exons (http://gsds.gao-lab.org/Gsds_about.php).

### Seed germination under water and salinity stress

Seed germination of WT and *dwfcp* was tested under salinity stress. Seeds were incubated in 50-ml Falcon tubes containing 25 ml of distilled water (control) or 200 mM NaCl for 16 h at 24°C in darkness under continuous shaking. After 16 h of imbibition, seeds were seeded in Petri plates between two filter papers moistened with the corresponding solution. The Petri plates with seeds were then incubated in a dark growth chamber at 24°C and 80% RH for 78 h. Two independent experiments were performed to study seed germination. Every 2 h for 80 h, germination seed was recorded. ImageJ® was used to compare root elongation from digital images taken 27 and 50 HAG. Before treatment, WT and mutant seeds were subjected to the same conditions since they were selected from BC_2_S_1_ seeds developed in the same fruit. The identification of WT and mutant seeds was done after genotyping for the causal mutation in the gene *CpDWF5* using individual DNA from radicles. The concentration of NaCl used for this assay was selected after analyzing the effect of different concentrations of NaCl (85, 150, 200, 250, and 300 mM) on the seed germination of the genetic background of the collection MUCU16 following the same protocol described (Supplemental Fig. S5).

### Seedling etiolation under control and salinity stress

Etiolation in darkness of WT and *dwfcp* seedlings was evaluated under 100 mM NaCl in two independent experiments. A total of 247 seeds from the segregating population BC_2_S_1_ were incubated in 50-ml Falcon tubes containing 25 ml of distilled water for 16 h at 24°C in darkness under continuous shaking. The seeds were then seeded in Petri dishes between two filter papers moistened with distilled water and incubated in a dark growth chamber at 24°C and 80% RH for 72 h. After this time, the germinated seeds were seeded in pots containing vermiculite and then randomly distributed in the growth chamber. The control and saline solutions were prepared in distilled water supplemented with nutrient solution (1 g/l), and the pH was adjusted to 5.8. 100 mM NaCl was added to the saline solution. The conductivities of the control and salt solutions were 1.725 and 11.573 dS/m, respectively. The pots with the germinated seeds were incubated in a growth chamber under the same conditions described for germination. After 72 h, the length and weight of hypocotyl and roots were measured in etiolated seedlings after extracting them from the pot and washing them to remove vermiculite. The identification of WT and mutant seeds was done after genotyping for the causal mutation in the gene *CpDWF5* using individual DNA from cotyledons. Apical shoot and root samples were collected to measure the relative expression of a set of salt stress-associated genes. The concentration of NaCl used for this assay was selected after analyzing the effect of different concentrations of NaCl (30, 60, 100, and 150 mM) on seedling growth of the genetic background of the mutant collection MUCU16 following the same protocol described (Supplemental Fig. S5).

### Statistical analyses

An analysis of variance (ANOVA) was performed in Statgraphic Centurion XVIII statistical software. The least significant difference (LSD) at a significance level of *P* ≤ 0.05 was used for multiple comparisons between genotypes and treatments.

## Acknowledgements

This work was supported by grants PID2020-118080RB-C21 and P20_00327 funded by the Spanish Ministry of Science and Innovation and the Junta de Andalucía. J.I.-M. and G.C. gratefully acknowledge the FPI and FPU Scholarship Program from the Spanish Ministry of Science and Innovation.

## Data Availability

The data underlying this article are available in the article and in its online supplementary material.

## Conflict of Interests

The authors have no conflicts of interest to declare.

## Supplementary Data

Supplementary data is available at *Horticulture Research* online.

## Supplementary Material

Web_Material_uhae050
