# Supplementary material for: A mutation in the brassinosteroid biosynthesis gene CpDWF5 disrupts vegetative and reproductive development and the salt stress response in squash (Cucurbita pepo)
Source: Hortic Res. 2024 Feb 23;11(4):uhae050. doi: 10.1093/hr/uhae050 (PMC11031414; doi:10.1093/hr/uhae050)
Supplement: Web_Material_uhae050 [file web_material_uhae050.zip › Figure S1.pdf]

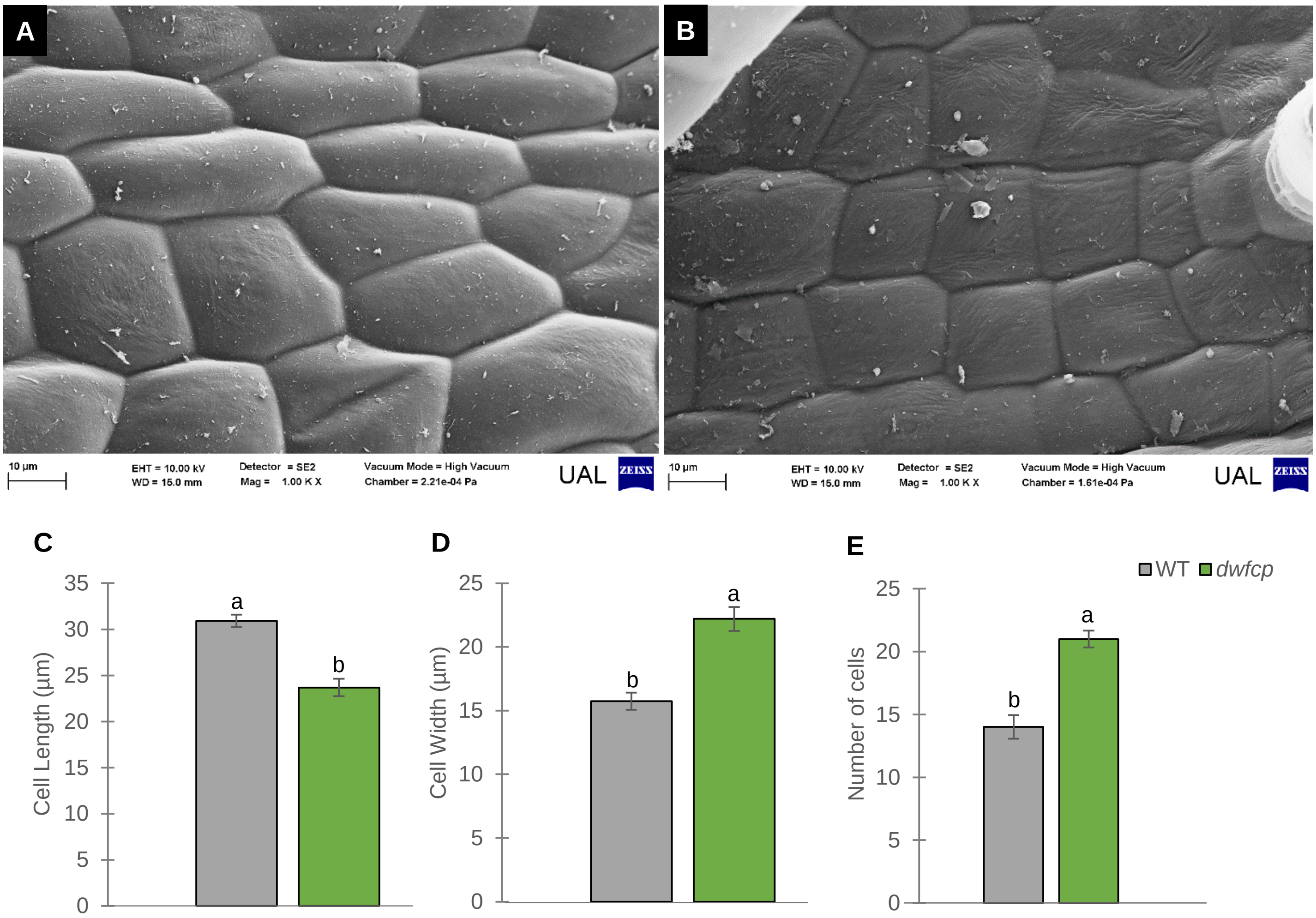

FIGURE S1 | Scanning electron microscopy (SEM) images of **(A)** WT and **(B)** *dwfcp* stems at T7 stage. Comparison of cells between WT and *dwfcp* stems. **(C)** Cell length, **(D)** width and **(E)** number of cells in longitudinal sections of WT and *dwfcp* stems. Error bars represent SE. Different letters indicate statistically significant differences ($p \leq 0.05$) between samples.
