# Supplementary material for: A mutation in the brassinosteroid biosynthesis gene CpDWF5 disrupts vegetative and reproductive development and the salt stress response in squash (Cucurbita pepo)
Source: Hortic Res. 2024 Feb 23;11(4):uhae050. doi: 10.1093/hr/uhae050 (PMC11031414; doi:10.1093/hr/uhae050)
Supplement: Web_Material_uhae050 [file web_material_uhae050.zip › Figure S3.pdf]

```
SlS7R-1      ---------------MVENKLVHSPLITYGSMLSLLSFTPPFVILMWYTNVHADGSILKT   45
SlS7R-2      ---------------MAESQLVHPPLFTYISMITLLTLVPPFVILMWYTNVHADGSVLQT   45
OsS7R        MAKPRASAAAAKAPASTPPKTVHSALVTYASMLSLLSLCPPFVILLWYTMVHADGSVVRA   60
AtS7R        -----------------MAETVHSPIVTYASMLSLLAFCPPFVILLWYTMVHQDGSVTQT   43
CpS7R        --------------MAEGSNTVHSPLVTYASMLSLLTLCPPFVIFLWYTMVHADGSVSQT   46
CmosS7R      --------------MAEGSNTVHSPLVTYASMLSLLTLCPPFVIFLWYTMVHADGSVSQT   46
CmaxS7R      --------------MAEGSNTVHSPLVTYASMLSLLTLCPPFVIFLWYTMVHADGSVSQT   46
CmS7R        --------------MAEGSNTVHSPFLTYVSMLSLLTLCPPFVILLWYTMVHADGSVSQT   46
CsS7R-X1     --------------MAEGSNTVHSPFLTYVSMLSLLTLCPPFVILLWYTMVHADGSVSQT   46
CsS7R-X2     -------------------------------------------------MVHADGSVSQT   11
                                                               ** ***: ::

SlS7R-1      FNHLRENG-LQGLIDIWPKPTAIAGKLIICYALFEAALQLLLPGKTVEGPISPTGHRPVY   104
SlS7R-2      YNYLKENG-LQGLIDIWPRPTAIAGKIIICYALFEATLQLLLPGKRVEGPISPTGHRPVY   104
OsS7R        YEHLREHGVLEGLKAIWPMPTMAAWKIIFGFGLFEAALQLLLPGKRFEGPVSPSGNVPVY   120
AtS7R        FGFFWENG-VQGLINIWPRPTLIAWKIIFCYGAFEAILQLLLPGKRVEGPISPAGNRPVY   102
CpS7R        MAYLYRNG-LRGFIDIWPPPSATAFKLIGSYAAFEAALQLLLPGKTVYGPISPTGNQPVY   105
CmosS7R      MAYLYHNG-LRGLIDIWPPPSATAFKLIGSYAAFEAALQLLLPGKTVYGPISPTGNQPVY   105
CmaxS7R      MAYLYHNG-LRGFIDIWPPPSATAFKLIGSYAAFEAALQLLLPGKTVYGPISPTGNQPVY   105
CmS7R        WNYLYQNG-FEGFINIWPSPSATACKIIGCYAAFEAALQLLLPGKRVLGPVSPTGNQPVY   105
CsS7R-X1     WNYLYQNG-FEGFINIWPSPSATACKIIGCYAAFEAALQLLLPGKRVLGPVSPTGNQPVY   105
CsS7R-X2     WNYLYQNG-FEGFINIWPSPSATACKIIGCYAAFEAALQLLLPGKRVLGPVSPTGNQPVY   70
               .: .:* ..*:  *** *:  * *:*  :. *** ******** . **:**:*: ***

SlS7R-1      KANGMAAYAVTLITYISLWWFGIFNPAIVYDHLGEIFSTLIFGSLVFCVLLYIKGHVAPS   164
SlS7R-2      KANGMAAYTVTLITYLSLWWFGIFNPTIVYDHLGEILSTLNFGSLIFCLFLYIKGHVAPS   164
OsS7R        KANGLQAYAVTLITYLSLWWFGIFNPAIVYDHLGEIYSALVFGSFVFCIFLYIKGHLAPS   180
AtS7R        KANGLAAYFVTLATYLGLWWFGIFNPAIVYDHLGEIFSALIFGSFIFCVLLYIKGHVAPS   162
CpS7R        KANGVAAYVVTLVTYIALWRFGIFNPTVVYDHLGEIFSALIIGSFIFCIVLYIKGCVAPS   165
CmosS7R      KANGVAAYVVTLVTYIALWWFGIFNPTVVYDHLGEIFSALIIGSFIFCIVLYIKGCVAPS   165
CmaxS7R      KANGVAAYVVTLVTYIALWRFGIFNPTVVYDHLGEIFSTLIIGSFIFCIVLYIKGCVAPS   165
CmS7R        KANGVAAYIVTLVTYVALWRFGIFDPAIVYDHLGEIFSTLIFGSFIFCILLYIKGHVAPS   165
CsS7R-X1     KANGVAAYIVTLLTYVALWRFGIFDPAIVYDHLGEIYSTLIFGSFIFCILLYIKGHVAPS   165
CsS7R-X2     KANGVAAYIVTLLTYVALWRFGIFDPAIVYDHLGEIYSTLIFGSFIFCILLYIKGHVAPS   130
             ****: ** *** **:.** ****:*::******** *:* :**::**:.***** :***

SlS7R-1      STDSGSSGNIIVDFYWGMELYPRIGKHFDIKVFTNCRFGMMSWAVLAVTYCIKQHEEYGR   224
SlS7R-2      STDHGSSGNIIVDYYWGMELYPRIGKHFDIKVFTNCRFGMISWGLLPITYCIKQYEEYGS   224
OsS7R        SSDSGSSGNVIIDFYWGMELYPRIGKHFDIKVFTNCRFGMMSWAVLAVTYCIKQYEMNGR   240
AtS7R        SSDSGSCGNLIIDFYWGMELYPRIGKSFDIKVFTNCRFGMMSWAVLAVTYCIKQYEINGK   222
CpS7R        SSDSGSSGNIIIDFYWGMELYPRIGKHFDIKVFTNCRFGMMSWAVLAVTYCIKQYEKNGT   225
CmosS7R      SSDSGSSGNIIIDFYWGMELYPRIGKHFDIKVFTNCRFGMMSWAVLAVTYCIKQYEKNGT   225
CmaxS7R      SSDSGSSGDIIIDFYWGMELYPRIGKHFDIKVFTNCRFGMMSWAVLAVTYCINQYEKNGT   225
CmS7R        SSDSGSSGNIILDFYWGMELYPRIGKHFDIKVFTNCRFGMMSWAVLAVTYCIKQYEKNGI   225
CsS7R-X1     SSDSGSSGNVILDFYWGMELYPRIGKHFDIKVFTNCRFGMMSWAVLAVTYCIKQYEKNGT   225
CsS7R-X2     SSDSGSSGNVILDFYWGMELYPRIGKHFDIKVFTNCRFGMMSWAVLAVTYCIKQYEKNGT   190
             *:* **.*::*:*:************ ************:**..* :****:*:*  *

SlS7R-1      VSDSMLVNTILMLVYVTKFFWWEAGYWNTMDIAHDRAGFYICWGCLVWVPSIYTSPGMYL   284
SlS7R-2      LSDSMLIHTIITLVYVTKFFWWEAGYWNTMDIAHDRAGFYICWGCLVFLPCMYTSPGMYL   284
OsS7R        VADSMLVNTALMLIYVTKFFWWESGYWCTMDIAHDRAGFYICWGCLVWVPSIYTSPGMYL   300
AtS7R        VSDSMLVNTILMLVYVTKFFWWEAGYWNTMDIAHDRAGFYICWGCLVWVPSVYTSPGMYL   282
CpS7R        IADSMLVNTALMLAYVTKFFWWEAGYWNTMDIAHDRAGFYICWGCLVWVPSVYTSPGMYL   285
CmosS7R      IADSMLVNTALMLAYVTKFFWWEAGYWNTMDIAHDRAGFYICWGCLVWVPSVYTSPGMYL   285
CmaxS7R      IADSMLVNTALMLAYVTKFFWWEAGYWNTMDIAHDRAGFYICWGCLVWVPSVYTSPGMYL   285
CmS7R        LADSMLVNTALMLVYVTKFFWWEAGYWNTMDIAHDRAGFYICWGCLVWVPSVYTSPGMYL   285
CsS7R-X1     LADSLLVNTALMLVYVTKFFWWEAGYWNTMDIAHDRAGFYICWGCLVWVPSVYTSPGMYL   285
CsS7R-X2     LADSLLVNTALMLVYVTKFFWWEAGYWNTMDIAHDRAGFYICWGCLVWVPSVYTSPGMYL   250
             ::**:*::* : * *********.*** *******************::*.:********

SlS7R-1      VKQPVNLGLQLSLYILVAGLLCIYINYDCDRQRQEFRRTNGKCTVWGKTPSKIVAAYTTT   344
SlS7R-2      VKHPVNLGPQLAISILVAGILCVYINYDCDRQRQEFRRTNGKALVWGKAPSKIVASYTTT   344
OsS7R        VNHPVNLGPQLALSILLAGILCIYINYDCDRQRQEFRRTNGKCSIWGKAPSKIVASYQTT   360
AtS7R        VNHPVELGTQLAIYILVAGILCIYINYDCDRQRQEFRRTNGKCLVWGRAPSKIVASYTTT   342
CpS7R        VNHPVNLGTQLAVYILVAGMLCIYINYDCDRQRQEFRRKNGKCLVWGKAPSKIVASYTTA   345
CmosS7R      VNHPVNLGTQLAVYILVAGMLCIYINYDCDRQRQEFRRKNGKCLVWGKAPSKIVASYTTT   345
CmaxS7R      VNHPVNLGIQLAVYILVAGMLCIYINYDCDRQRQEFRRKNGKCLVWGKAPSKIVASYTTT   345
CmS7R        VNHPVNLGTQLAVSILVAGILCIYINYDCDRQRQEFRRKNGKRLVWGKAPSKIVASYTTT   345
CsS7R-X1     VNHPVNLGTQLAVSILVAGILCIYINYDCDRQRQEFRRKNGKCLVWGKAPSKIVATYTTT   345
CsS7R-X2     VNHPVNLGTQLAVSILVAGILCIYINYDCDRQRQEFRRKNGKCLVWGKAPSKIVATYTTT   310
             *::**:** **:: **:**:**.*************** .***  :**::******:* *:

SlS7R-1      SGEKKTSLLLTSGWWGLARHFHYVPEILAAFFWSVPALFNHFIPYFYVIFLIILLLDRAK   404
SlS7R-2      TGETKTSILLTSGWWGLSRHFHYVPEILASFFWSVPALLNHFMPYIYVIYLTGLLLDRAK   404
OsS7R        NGETKSSLLLTSGWWGLSRHFHYVPEILSAFFWTVPALFDHFLPYFYVIFLTILLFDRAK   420
AtS7R        SGETKTSLLLTSGWWGLARHFHYVPEILSAFFWTVPALFDNFLAYFYVIFLTLLLFDRAK   402
CpS7R        SGETKTSLLLTSGWWGLARHFHYVPEILAAFFWTVPALFNHFLPYFYVIFLTILLVDRAK   405
CmosS7R      SGETKTSLLLTSGWWGLARHFHYVPEILAAFFWTVPALFNHFLPYFYVIFLTILLVDRAK   405
CmaxS7R      SGETKTSLLLTSGWWGLARHFHYVPEILAAFFWTVPALFNHFLPYFYVIFLTILLVDRAK   405
CmS7R        SGETRTSLLLTSGWWGLARHFHYVPEILAAFFWTVPALFHHFLPYFYVIFLTILLFDRAK   405
CsS7R-X1     TGETRTSLLLTSGWWGLARHFHYVPEILAAFFWTVPALFHHFLPYFYVIFLTILLFDRAK   405
CsS7R-X2     TGETRTSLLLTSGWWGLARHFHYVPEILAAFFWTVPALFHHFLPYFYVIFLTILLFDRAK   370
             .**.::*:*********:**********::***:****.::*: *:***:*  **.****

SlS7R-1      RDDDRCKAKYGKYWKLYCEKVPYRVIPGIY   434
SlS7R-2      RDDERCKSKYGKYWKKYCEKVPYRVVPGIY   434
OsS7R        RDDDRCSSKYGKYWKMYCNKVPCRVIPGIY   450
AtS7R        RDDDRCRSKYGKYWKLYCEKVKYRIIPGIY   432
CpS7R        RDDDRCRSKYGKYWKLYCEKVPYRIIPGIC   435
CmosS7R      RDDDRCRSKYGKYWKLYCEKVPYRIIPGIC   435
CmaxS7R      RDDNRCRSKYGKYWKLYCEKVPYRIIPGIC   435
CmS7R        RDDDRCRSKYGKYWKLYCEKVPCKIIPGIY   435
CsS7R-X1     RDDDRCRSKYGKYWKLYCEKVPYKIIPGIY   435
CsS7R-X2     RDDDRCRSKYGKYWKLYCEKVPYKIIPGIY   400
             ***:** :******* **:**  :::***
```

FIGURE S3 | **Multiple sequences alignment of CpS7R and its homologous proteins.**
