# Supplementary material for: A mutation in the brassinosteroid biosynthesis gene CpDWF5 disrupts vegetative and reproductive development and the salt stress response in squash (Cucurbita pepo)
Source: Hortic Res. 2024 Feb 23;11(4):uhae050. doi: 10.1093/hr/uhae050 (PMC11031414; doi:10.1093/hr/uhae050)
Supplement: Web_Material_uhae050 [file web_material_uhae050.zip › Figure S4.pdf]

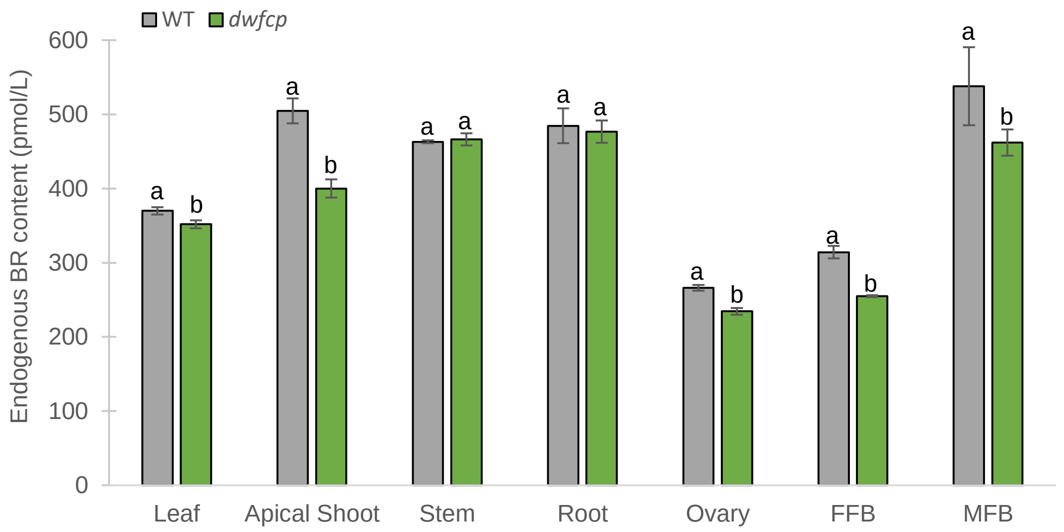

FIGURE S4 | **Comparison of endogenous BR content in leaf, apical shoot, stem, root, ovary, FFB and MFB between WT and *dwfcp*.** FFB, female floral bud excluding the ovary; MFB, male floral bud. Error bars represent SE. Different letters indicate statistically significant differences ($p \leq 0.05$) between samples.
