# Supplementary material for: A mutation in the brassinosteroid biosynthesis gene CpDWF5 disrupts vegetative and reproductive development and the salt stress response in squash (Cucurbita pepo)
Source: Hortic Res. 2024 Feb 23;11(4):uhae050. doi: 10.1093/hr/uhae050 (PMC11031414; doi:10.1093/hr/uhae050)
Supplement: Web_Material_uhae050 [file web_material_uhae050.zip › Figure S5.pdf]

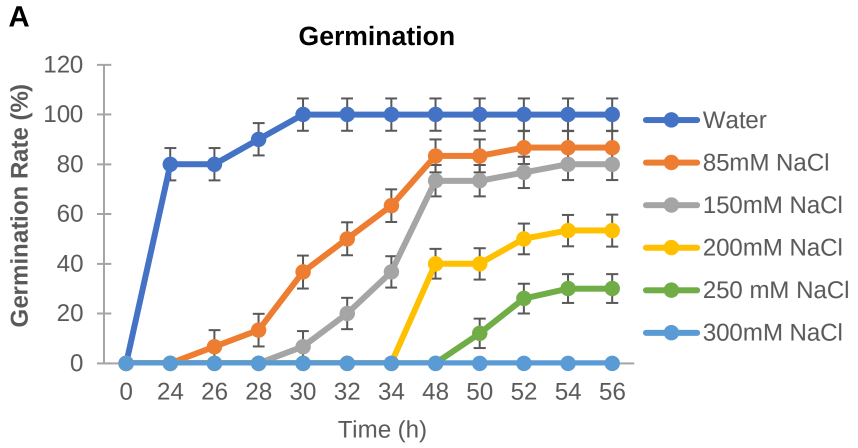

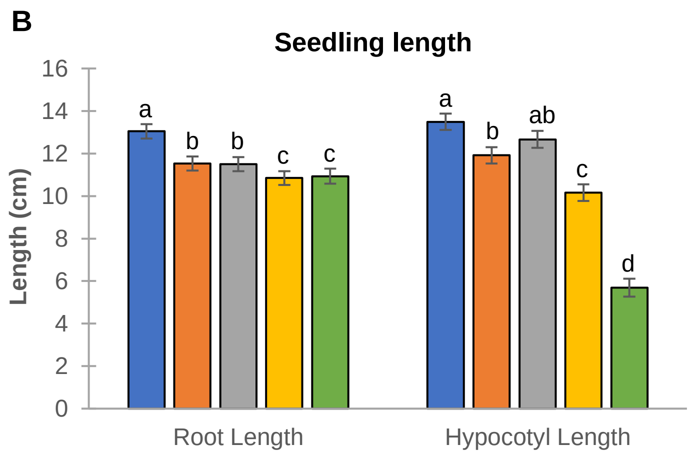

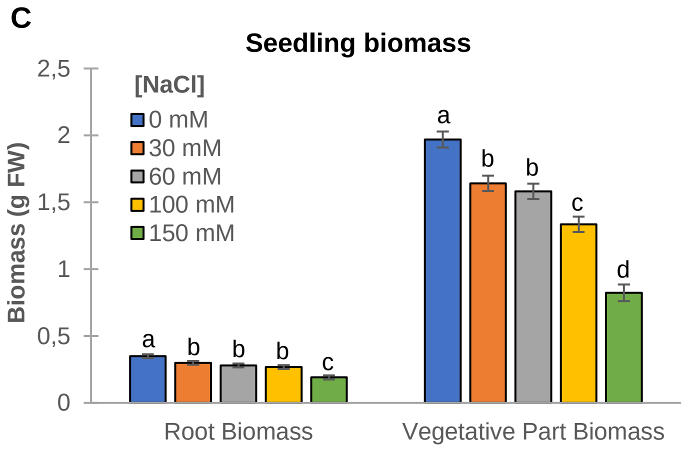

FIGURE S5 | Dose-response relationship between NaCl stress and germination and seedling growth in line MUCU16. (A) Effect of different concentrations of NaCl on the germination rate of MUCU16 over time. The seeds were imbibed for 16 h at 24ºC and then allowed to germinate between two filter papers soaked in the same solution. **(B-C)** Effect of different concentrations of NaCl on root and hypocotyl length (B) and biomass (C) of seedlings grown in darkness for 72 h. Error bars represent SE. Different letters indicate statistically significant differences ($p < 0.05$) between samples.
