# Supplementary material for: A mutation in the brassinosteroid biosynthesis gene CpDWF5 disrupts vegetative and reproductive development and the salt stress response in squash (Cucurbita pepo)
Source: Hortic Res. 2024 Feb 23;11(4):uhae050. doi: 10.1093/hr/uhae050 (PMC11031414; doi:10.1093/hr/uhae050)
Supplement: Web_Material_uhae050 [file web_material_uhae050.zip › Table S1.docx]

**Table S1 |** Inheritance of *dwfcp* mutant in backcrossing and selfing generation.

| Generation | WT | *dwfcp* | Expected segregation | X_C_^2^ | p-value |
| --- | --- | --- | --- | --- | --- |
|  | **Number of plants** | | | | |
| BC_1_ | 32 | - | 1:0 | - | - |
| BC_1_S_1_ | 226 | 72 | 3:1 | 0.42 | ˃ 0.05 |
| BC_2_S_1_ | 234 | 75 | 3:1 | 0.39 | ˃ 0.05 |
