# Supplementary material for: A mutation in the brassinosteroid biosynthesis gene CpDWF5 disrupts vegetative and reproductive development and the salt stress response in squash (Cucurbita pepo)
Source: Hortic Res. 2024 Feb 23;11(4):uhae050. doi: 10.1093/hr/uhae050 (PMC11031414; doi:10.1093/hr/uhae050)
Supplement: Web_Material_uhae050 [file web_material_uhae050.zip › Table S2.docx]

**Table S2 |** List of primer sequences used for qRT-PCR analysis and sequencing *CpDWF5* gene.

| Genes | Forward Primer | Reverse Primer |
| --- | --- | --- |
| Expression primers for *CpDWF5* and reference genes *CpEF1α* and *Cp18S* | | |
| *CpDWF5* | CTGTACTGCGAGAAAGTGCCT | ATGAAGTGAAGCGAGCGAGC |
| *CpEF1α* | CGTCAAGAAGAAATAAGCCA | CTACTACGAGAGAGAGAGCCG |
| *Cp18S* | CCGTTGCTCTGATGATTCATGA | GTTGATAGGGCAGAAATTTGAATGAT |
| Expression primers for genes associated with salt tolerance | | |
| *CpNCED2/5A* | TCTGGCTCAAGAACAACAGC | GTGGTACGGCAAATCGTCTT |
| *CpPP2C-A* | TCAGAGCTGCACGCCGC | GAATCTTTGCAAAACAAGGAACAG |
| *CpCYP707A2* | CGGCGACAAAGTGGAGAAAA | CGAACTCCACCTCTTCCACT |
| *CpKEA4-2A* | CCCCTACCGTCTCTCTTCCAC | AAGATGAAAATGCGGACAGG |
| *CpKUP6-1B* | TTCATCCGATCCGAGAAAAC | GATCGGCAAAGAACACGATT |
| *CpNHK1-3B* | CACTCAACTGATCGGGAGGT | CAAATGAGAGCGTTGCAAAA |
| *CpCRCK2B* | GCTCATGCTATCACCTATCTTCA | TAACTTGGGTCGAGACATGC |
| Sequencing primers | | |
| *CpDWF5*  (from nucleotide 98 to 896) | GCCTCAAAAATGGCGGAAGGA | ACCAGCTCTATCATGCGCTA |
| *CpDWF5*  (from nucleotide 642 to 1424) | TCTACTGGGGCATGGAGTTG | GAAGCCGTATTAGCATATTCCAGG |
