# Supplementary material for: A mutation in the brassinosteroid biosynthesis gene CpDWF5 disrupts vegetative and reproductive development and the salt stress response in squash (Cucurbita pepo)
Source: Hortic Res. 2024 Feb 23;11(4):uhae050. doi: 10.1093/hr/uhae050 (PMC11031414; doi:10.1093/hr/uhae050)
Supplement: Web_Material_uhae050 [file web_material_uhae050.zip › Table S3.docx]

**Table S3 |** List of protein sequences used in sequence alignment and phylogenetic analysis.

| Species | Accession numbers | Protein | Number of aa |
| --- | --- | --- | --- |
| Multiple alignment | | | |
| *Arabidopsis thaliana* | NP_175460.1 | AtS7R | 431 |
| *Solanum lycopersicum* | NP_001353044.1 | SlS7R-1 | 434 |
| *Solanum lycopersicum* | NP_001353043.1 | SlS7R-2 | 434 |
| *Oryza sativa* | XP_015627380.1 | OsS7R | 450 |
| *Cucurbita moschata* | XP_022964233.1 | CmS7R | 435 |
| *Cucurbita maxima* | XP_022999960.1 | CmS7R | 435 |
| *Cucumis melo* | XP_008462206.2 | CmS7R | 435 |
| *Cucumis sativus* | XP_011659586.1 | CsS7R-X1 | 435 |
| *Cucumis sativus* | XP_031744858.1 | CsS7R-X2 | 400 |
| *Cucurbita pepo* | XP_023513993.1 | CpS7R | 435 |
| Other sterol reductases used in phylogenetic tree | | | |
| *Arabidopsis thaliana* | NP_566975.1 | AtS14R | 369 |
| *Solanum lycopersicum* | NP_001238799.2 | SlS14R | 369 |
| *Cucurbita pepo* | XP_023526298.1 | CpS14R | 369 |
